# “I DON’T USE ANYTHING SPECIAL”: DIETARY SELF-MANAGEMENT AMONG BLIND AND VISION-IMPAIRED PERSONS WITH TYPE 2 DIABETES

**DOI:** 10.1093/geroni/igae098.2170

**Published:** 2024-12-31

**Authors:** Emily Nicklett, Julia Crowley, Rene Perez, Vanessa Fears, Beatriz Morales Hernandez, Yesenia Burgos-Melendez

**Affiliations:** The University of Texas San Antonio, San Antonio, Texas, United States; Vibrant Works, San Antonio, Texas, United States; Vibrant Works, San Antonio, Texas, United States; The University of Texas San Antonio, San Antonio, Texas, United States; The University of Texas San Antonio, San Antonio, Texas, United States; The University of Texas San Antonio, San Antonio, Texas, United States

## Abstract

Diabetic retinopathy, a common complication of type 2 diabetes (T2D), is a leading cause of blindness and vision impairment (BVI) among middle-aged and older adults. Diabetes self-management is critical for maintaining eyesight and reducing the risk of further diabetes-related complications; however, those with BVI likely face additional challenges in managing diabetes, including following a diabetes-friendly diet. This study explores barriers and facilitators to maintaining a diabetes-friendly diet among BVI persons with T2D and examines how these experiences may influence their dietary behaviors. In collaboration with Vibrant Works—a community-based organization in San Antonio, Texas offering employment, independent living, and well-being services to the blind and low-vision community—phone-based questionnaires were fielded in January-March 2023 (n=18, ages 42-70). The questionnaires included validated measures of dietary adherence, followed by an open-ended, semi-structured interview about challenges and strategies for diabetes self-management. Quantitative data were entered directly into a database and qualitative data were transcribed and coded with themes emerging directly from the data. Six themes emerged that characterize barriers and facilitators to dietary behaviors: (a) vision status and comorbidities; (b) prior knowledge and experience in food preparation; (c) habits and routines; (d) access/accessibility (i.e., transportation, BVI-friendly cooking tools, accessibility of food stores; (e) support (i.e., social and tangible support); and (f) neighborhood and community factors (i.e., walkability, sidewalk quality, food store availability). Findings from this study can inform more equitable strategies for dietary self-management among populations experiencing both T2D and vision loss through direct practice, future research, and policy approaches.

